# The Advantages and Accidents of Artificial Anæsthesia

**Published:** 1878-12

**Authors:** 


### BIBLIOGRAPHICAL.

The Advantages and Accidents of Artificial Anæsthesia. By Lawrence Turnbull, M. D., Ph. G. Philadelphia—Lindsay & Blackiston.

This is a plain and practical volume of some 200 pages, on the subject as above, and is an exceedingly interesting contribution to the literature of the times. The writer comes to his work with all the well known Northern prejudices in favor of ether and against chloroform, and although striving to be fair, shows throughout the entire discussion of the subject that it is difficult to shake off the influence of preconceived bias in favor of ether. With regard to chloroform, Dr. Turnbull says:

"By a reference to the recent cases of deaths from this agent I am fully satisfied that no amount of care or precaution, or mode of administration, or amount inhaled, will prevent in certain cases the fatal result, and yet physicians and others will resort to the use of chloroform, on account of its pleasant taste and odor, rapidity of action, cost, and comparative bulk. I have given its advocates every opportunity to state where, when, and how it can be given with safety, omitting nothing through prejudice or favor, having in my practice resorted to its use before being aware of the great risk incurred to every patient."

But we are far from finding fault with this book, which no one can read without profit; and Dr. Turnbull has done a real service to the profession in writing it. The various anæsthetics are fully treated of, and a Section devoted to nitrous oxide gas. A description of the inhalers in use for administering anæsthetics, with a number of illustrations, add to the value of the book; besides will be found formulæ for local anæsthetics, &c.

We have not noticed in reading the book in question that mention is made of the suggested, and to some extent practised, use of oxygen gas in conjunction with nitrous oxide gas as an anæsthetic. The writer tried this combination (one-fifth oxygen) some years ago with seemingly favorable results. There was a lessening of the stertor, and lividity, and the unhappy physical symptoms generally present with the administration of that agent; though the unconscious condition seemed lessened in proportion. Pure oxygen gas itself has also been used, and the prediction is made by Dr. J. Lynk, (see Dr. Turnbull's book) that alcohol will, by the next decade, rank first as an anæsthetic.

Those who wish a very fair resumé of the progress of anæsthesia, and who desire to have at hand a practical and useful book on the actual manipulation of these agents, cannot dispense with this little book; and the old practitioner as well can read it with profit. H.

